# Altitude and latitude have different effects on population characteristics of the widespread plant *Anthyllis vulneraria*

**DOI:** 10.1007/s00442-021-05030-6

**Published:** 2021-10-02

**Authors:** Laura Daco, Guy Colling, Diethart Matthies

**Affiliations:** 1 Department of Biology, University of Marburg, Karl-von-Frisch-Str. 8, 35043 Marburg, Germany; 2 Musée national d’histoire naturelle, 25 rue Münster, L-2160 Luxembourg, Luxembourg; 3 Fondation Faune-Flore, 24 rue Münster, L-2160 Luxembourg, Luxembourg

**Keywords:** Abundant centre model, Climate change, Environmental gradients, Herbivory, Seed set

## Abstract

Widespread plants may provide natural models for how population processes change with temperature and other environmental variables and how they may respond to global change. Similar changes in temperature can occur along altitudinal and latitudinal gradients, but hardly any study has compared the effects of the two types of gradients. We studied populations of *Anthyllis vulneraria* along a latitudinal gradient from Central Europe to the range limit in the North and an altitudinal gradient in the Alps from 500 m to the altitudinal limit at 2500 m, both encompassing a change in annual mean temperature of c. 11.5 °C. Plant size and reproduction decreased, but plant density increased along both gradients, indicating higher recruitment and demographic compensation among vital rates. Our results support the view that demographic compensation may be common in widespread species in contrast to the predictions of the abundant centre model of biogeography. Variation in temperature along the gradients had the strongest effects on most population characteristics, followed by that in precipitation, solar radiation, and soil nutrients. The proportion of plants flowering, seed set and seed mass declined with latitude, while the large variation in these traits along the altitudinal gradient was not related to elevation and covarying environmental variables like annual mean temperature. This suggests that it will be more difficult to draw conclusions about the potential impacts of future climate warming on plant populations in mountains, because of the importance of small-scale variation in environmental conditions.

**Supplementary Information:**

The online version contains supplementary material available at 10.1007/s00442-021-05030-6.

## Introduction

Temperature is an important determinant of plant physiology and distribution (Woodward 1987; De Frenne et al. 2013). Climatic factors such as low temperatures and short growing seasons typically constrain plant growth and cause absolute limits to plant growth in arctic and alpine environments (Woodward 1987; Körner 2003; Halbritter et al. 2013). According to the altitude-for-latitude temperature model, similar changes in annual mean temperature of c. 5 °C occur over 1000 m of altitude and 1000 km of latitude in the temperate zone (Jump et al. 2009). Studies of population traits along gradients of altitude and latitude with comparable changes in temperature provide an opportunity to study the influence of temperature and other environmental factors on plant population characteristics and structure. The study of natural variation in ecologically important traits is crucial to further increase our knowledge of the mechanisms involved in the distribution and abundance of plant species (Jump et al. 2009).

Global warming is expected to affect the range and abundance of species in the future (Walther 2003; Thomas 2010; Swab et al. 2015). The global mean surface temperature is projected to increase during this century by up to 4.8 °C (IPCC 2014). An improved understanding of how climatic conditions influence plants is important for assessing the response of plants to global change and to conserve threatened plant species under changing climatic conditions. Plant species with populations occurring along strong gradients in altitude or latitude and corresponding strong changes in temperature may provide natural models for how population processes change with temperature (Halbritter et al. 2013).

However, altitudinal and latitudinal gradients differ in a number of factors including atmospheric pressure, precipitation, solar radiation, and soil nutrients (Körner 2007; De Frenne et al. 2013). The response of species across the two types of gradients may thus be expected to differ in potentially important aspects. While many studies have investigated different processes in plant populations along either altitudinal (Totland 2001; Pellissier et al. 2010; Rasmann et al. 2014) or latitudinal gradients (Sagarin et al. 2006; Moeller et al. 2017), there are hardly any studies that have compared the effect of the two types of gradients on populations of a single species (Siefert et al. 2015). Moreover, most studies did not try to disentangle the effects of temperature on plants from those of other environmental factors (De Frenne et al. 2013).

The aim of this study was to compare populations of *Anthyllis vulneraria* L. (Fabaceae) along two temperature gradients of c. 10 °C, a latitudinal gradient of c. 2400 km from Central Europe to northern Norway and three altitudinal gradients of c. 2000 m in the European Alps. We analysed climatic conditions using data from Worldclim (Fick and Hijmans 2017), soil nutrients and site productivity and investigated characteristics of the populations including their size and density, size-related plant traits, reproduction, pre-dispersal seed predation, and population structure. The altitudinal gradient spanned the species' range from lowland sites to populations at the altitudinal limit and the latitudinal gradient ranged from the geographical centre of the distribution to the northern distribution limit. These gradients allowed us to test some of the predictions of the abundant centre model (ACM; Brown 1984; Sagarin and Gaines 2002) of biogeography. The ACM predicts that the abundance and performance of species at the geographical periphery of their distribution area are lower than those at the centre of their range where environmental conditions are assumed to be most suitable (Abeli et al. 2014; Pironon et al. 2017).

We address the following questions: (1) How do population characteristics of *A. vulneraria* vary along the two gradients and do altitude and latitude have different effects? (2) Which environmental variables in addition to temperature are important predictors of population characteristics along altitudinal and latitudinal gradients? (3) Are characteristics of the populations along both gradients in line with the predictions of the ACM?

## Material and methods

### Study species

*Anthyllis vulneraria* is a very polymorphic biennial or perennial occurring in nutrient-poor calcareous grasslands and screes all across Europe, the Mediterranean Basin and the Caucasus (GBIF.org 2019) from sea level up to 3000 m a.s.l. (Conert 1975). In total, 24 infraspecific taxa have been described in Europe (Cullen 1968). However, a phylogenetic study based on ITS sequences and chloroplast microsatellites showed that all subspecies of *A. vulneraria* clustered together and thus did not support the traditional taxonomic subdivisions based on morphology (Nanni et al. 2004). Moreover, AFLP variance in a study of eight taxa of *A. vulneraria* did not support recognizing intraspecific taxa of *A. vulneraria* at the species or subspecies level (Köster et al. 2008). In contrast to earlier studies which argued that subspecies do not interbreed due to the predominantly autogamous reproductive system (Couderc 1971; Couderc and Gorenflot 1978) more recent studies based on molecular markers found that there is considerable gene flow among and within populations (Honnay et al. 2006; Van Glabeke et al. 2007; Kesselring et al. 2015) indicating that the populations are not predominantly selfing. In the present study, we will therefore not distinguish between putative subspecies a priori to capture a large amount of variation over the whole study area. The basal leaves of *A. vulneraria* form rosettes and the flowers are grouped into flowerheads with four to more than 20 flowers. Plants usually start flowering in the second year after germination, but flowering can be delayed for up to eight years (Erschbamer and Retter 2004). Most plants are monocarpic but some may flower more than once (Sterk 1975). Flowers are pollinated by Hymenoptera (Couderc and Gorenflot 1978) and produce a single seed per fruit. An important specialist herbivore of *A. vulneraria* are the caterpillars of *Cupido minimus* (Lepidoptera) which feed on the developing seeds (Krauss et al. 2004). Although *A. vulneraria* is not considered to be endangered in most parts of its distribution, it has strongly declined in some areas in the last decades, e.g. in NE Germany (Jansen et al. 2019).

### Altitudinal and latitudinal gradients

We studied the influence of environmental conditions on *A. vulneraria* in 40 populations along an altitudinal and a latitudinal gradient. The length of the two gradients was chosen to correspond to a change of 11.5 °C in annual mean temperature. The latitudinal gradient ranged from the centre of the distribution in Central Europe (46.4 °N) over 2400 km to the northern distribution limit in Scandinavia (68.1°N) and the altitudinal gradient from valley populations in the Alps at 500 m to the altitudinal limit at 2500 m a.s.l. To account for potential regional climatic differences, the altitudinal gradient was replicated in three different alpine regions in France, Switzerland and Austria.

### Field data

**Fig. 1:**
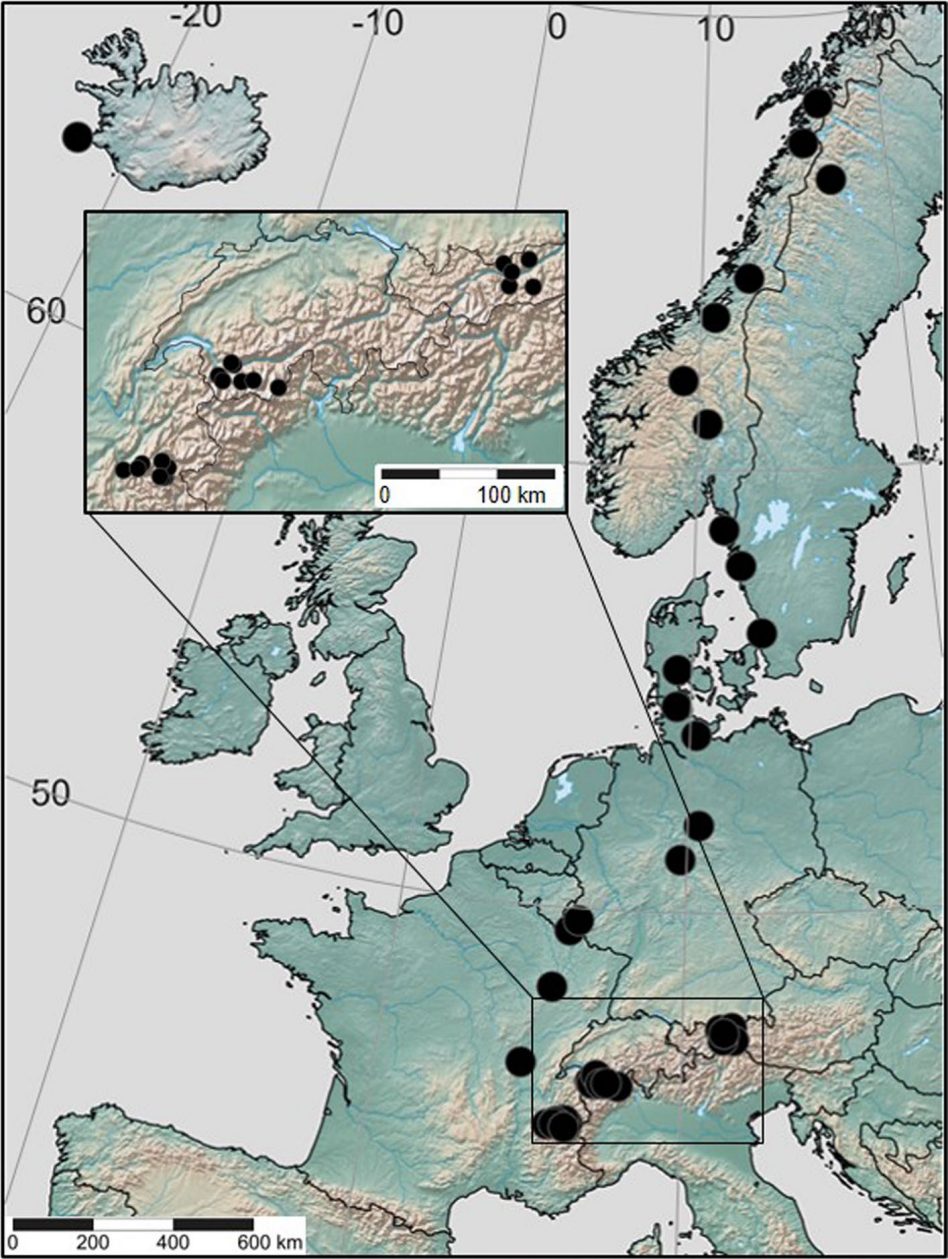
Map of the *Anthyllis vulneraria* study sites in Europe. The inset shows the locations of the populations of the altitudinal gradient in France, Switzerland and Austria

Based on information gathered from local botanists and the GBIF database (GBIF.org 2019), we sampled 20 *A. vulneraria* populations each along the altitudinal and the latitudinal gradients (Fig. 1; Supplementary Material Table S1). A population was defined as a group of plants separated by at least 100 m from the next conspecific plants. During summer 2015, we recorded at each site the altitude above sea level, latitude and longitude with a GPS (eTrex 20, Garmin Ltd.), site exposition as absolute deviation from the north with a compass and inclination with a clinometer. Populations were sampled at a similar phenological state, i.e. southern and lowland populations were sampled first. In small populations, all plants of *A. vulneraria* were counted. In large populations, the population size was estimated from counts in parts of the total area. To estimate site productivity, we sampled in each population the above-ground biomass in two randomly chosen plots (25 cm × 25 cm) in which *A. vulneraria* was present. The vegetation was clipped at 5 cm above soil level, dried at 60 °C for 48 h to constant mass and weighed. At each site, we took at least three random samples of a total of at least 300 g of mineral soil. Soil organic carbon was determined by subtracting the inorganic carbon content measured with phosphoric acid from the total carbon content determined by dry combustion at 1200 °C with a Multi EA 4000 (Analytik Jena, Jena, Germany). Total nitrogen content was determined by dry combustion at 900 °C with an elemental analyzer (Vario MAX cube, Elementar, Langenselbold, Germany). The pH was measured in a 0.01 M solution of CaCl_2_ with a SP2000 pH soil analyser (Skalar Analytical, Breda, Netherlands). Phosphorus (P_2_O_5_) and potassium (K_2_O) were extracted with a 0.05 M calcium lactate and acetate solution and the contents were dosed by inductively coupled plasma optical emission spectrometry (Agilent 725 ICP-OES Radial, Santa Clara, USA).

In each population, we selected one plot of 1 m × 1 m in an area which appeared to have the highest density of *A. vulneraria* and two further plots located at random but with *A. vulneraria* present. In each plot, we counted the number of flowerheads of *A. vulneraria* and of flowering plants and calculated the mean number of flowerheads per plant and the mean per population. In the three plots, we determined the sum of the number of vegetative and of flowering *A. vulneraria* individuals and calculated the proportion of flowering plants per population.

In each population we collected all flowerheads from 20 fruiting plants along a 20 m long transect. We determined the height for each of these plants. In the laboratory, we determined for each mother plant the number of healthy seeds (green and large), fully developed but damaged seeds with clear signs of herbivory, and aborted seeds (small, brown or light green). We calculated seed set per population as [number of intact and damaged seeds collected/total number of seeds including aborted], and the proportion of damaged seeds as [sum of damaged seeds/total number of seeds]. The presence of eggs of *Cupido minimus* in the collected flowerheads was recorded for each population. For each mother plant, the mean mass of healthy seeds was determined and then averaged to calculate mean seed mass per population.

### Bioclimatic variables

For each population, we downloaded three climatic variables from the WorldClim database Version 2.0 (Fick and Hijmans 2017) in a 30 arc-seconds (1 km^2^) resolution: annual mean temperature, annual precipitation, and solar radiation.

### Statistical analyses

If not stated otherwise, all statistical analyses were carried out using IBM SPSS Statistics for Windows, version 25.0 (IBM Corp., Armonk, N.Y., USA). To study the environmental changes with latitude and altitude, we analysed the relationships between altitude or latitude and annual mean temperature, annual precipitation, solar radiation, standing biomass, soil organic carbon content, nitrogen, phosphorus, and potassium content with linear regressions.

The effects of altitude and latitude on population means of morphological and reproductive traits were tested with general and generalized linear models (GLMs) in R 3.5.2 (R Core Team 2018). These analyses investigated also whether mean plant performance declined continuously from the centre of the distribution to the range margins at high latitudes and altitudes as predicted by the abundant centre model. The GLMs for proportion data (e.g. proportion flowering, seed set, seeds damaged) were calculated with a logit link and a quasibinomial error distribution (see Crawley 2009). For the altitudinal gradient, the effects of the three regions within the Alps and the linear and quadratic effects of altitude as well as the interaction between the region and the linear and the quadratic terms were tested. The quadratic term was integrated to check for a potential mid-elevational maximum along the gradient. Similarly, linear and quadratic effects of latitude were also tested; however, the quadratic terms were never significant. Models were simplified by dropping non-significant terms (*P* > 0.05). For proportion data, McFadden’s Pseudo *r*^*2*^ was calculated as one minus the ratio between the log-likelihood of the model of interest and the log-likelihood of the null model. When region had a significant effect (only in the case of maximum density), the *r*^*2*^ of the model for each region was calculated by correlating the predicted values from the overall model and the observed values. To study the possible effects of altitude or latitude, population size and maximum plant density on the presence of eggs of *Cupido minimus,* we carried out separate logistic regressions. Due to overdispersion, we used quasibinomial error distributions in the GLMs. We compared the strength of the relationships of mean values of population traits and altitude with the relationship of the same traits with latitude with *Z*-tests using the R-package cocor (Diedenhofen and Musch 2015).

We performed a principal component analysis (PCA) based on correlations with soil organic carbon, total nitrogen and phosphorus (P_2_O_5_) contents to characterize soil conditions at the study sites. The resulting PCA factor (PC Soil nutrients) explained 77.8% of the total variance of the component variables and was used in multiple regressions as an explanatory environmental variable. Factor loadings were 0.96 for *N*_tot_, 0.79 for P_2_O_5_, and 0.89 for *C*_org_. To elucidate which environmental variables might be responsible for the observed relationships between certain population characteristics and latitude or altitude, we performed (generalized) linear models with normal or quasibinomial errors relating these characteristics to the following explanatory variables: annual mean temperature, annual precipitation, solar radiation, PC Soil nutrients, standing biomass (see Supplementary Material Table S2). For each plant trait we calculated all possible models and their Akaike information criteria (AICc) or the quasi AICc (QAICc; for proportion data) using the function *dredge* of the R-package MuMIn (Barton 2019). To assess the importance of the individual predictors we averaged all possible models including these predictors (conditional average, Burnham and Anderson 2002) and derived importance values. In addition, we selected the model with the lowest AICc or QAICc. The *r*^*2*^ of those models was calculated by correlating the predicted and the observed values for the Gaussian data and by calculating Mc Fadden’s pseudo *r*^2^ in the case of proportion data. We calculated standardized regression coefficients for the variables in these models. Standardized regression coefficients for the GLMs for proportion data were calculated using the latent-theoretical method (Grace et al. 2018).

In the analyses, data for population size, maximum plant density and mean number of flowerheads per plant were log-transformed prior to analysis to achieve normally distributed residuals and homoscedasticity. Population AAt5 was excluded from most of the analyses due to missing data (except for population size, max. plant density, seed set, seeds damaged and seed mass).

## Results

### Habitat characteristics and gradients

Most study sites had a southern exposition (median = 114.5° deviation from north) indicating that *A. vulneraria* prefers well-exposed habitats (Supplementary Material Table S3). *Anthyllis vulneraria* was present at both level sites and slopes of up to 103% inclination. Standing biomass was generally low (median = 96.1 g/m^2^) indicating little competition for light. Soils were slightly acidic to neutral (median pH = 7.1) and the nutrient content was generally low (medians: *N*_tot_ = 0.2%, P_2_O_5_ < 3.0 mg/100 g soil, K_2_O = 8.0 mg/100 g soil, *C*_org_ = 3.2%). The range of annual mean temperatures at the study sites was similar for both the altitudinal (− 0.4 to 10.8 °C) and the latitudinal gradients (− 1.2 to 10.8 °C). Annual mean temperature decreased with both increasing altitude and latitude (Table 1, Supplementary Material Fig. S1).

**Table 1:** Correlations between altitude and various habitat characteristics for the 20 altitudinal populations and latitude and various habitat characteristics for the 20 latitudinal gradient populations separately. (*)*P* < 0.1; *P < 0.05; **P < 0.01; ***P < 0.001. ^a^Sample size *N* = 19

		Altitude	Latitude
Habitat variable		*r*	*r*
Annual mean temperature (°C)		− 0.98***	− 0.87***
Annual precipitation (mm)		0.63**	0.28
Solar radiation (kJ/m^2^ day)	All sites	0.33	− 0.95***
	France	0.99***	
	Switzerland	0.94**	
	Austria	0.82(*)	
Standing biomass (g/m^2^)		− 0.40(*) ^a^	0.36
Organic carbon soil content (%)		0.06^a^	− 0.49*
Nitrogen soil content (%)		0.14^a^	− 0.57**
Phosphorus soil content (mg/100 g soil)		0.17^a^	− 0.48*
K_2_O soil content (mg/100 g soil)		− 0.07^a^	− 0.33

Several other environmental variables changed along the gradients of altitude and latitude (Table 1). With increasing altitude precipitation increased and standing biomass decreased, while with increasing latitude solar radiation, soil N, P and organic C decreased. Potassium (K) soil content did not vary along the gradients.

### Population characteristics and mean plant traits along the altitudinal and latitudinal gradients

**Fig. 2:**
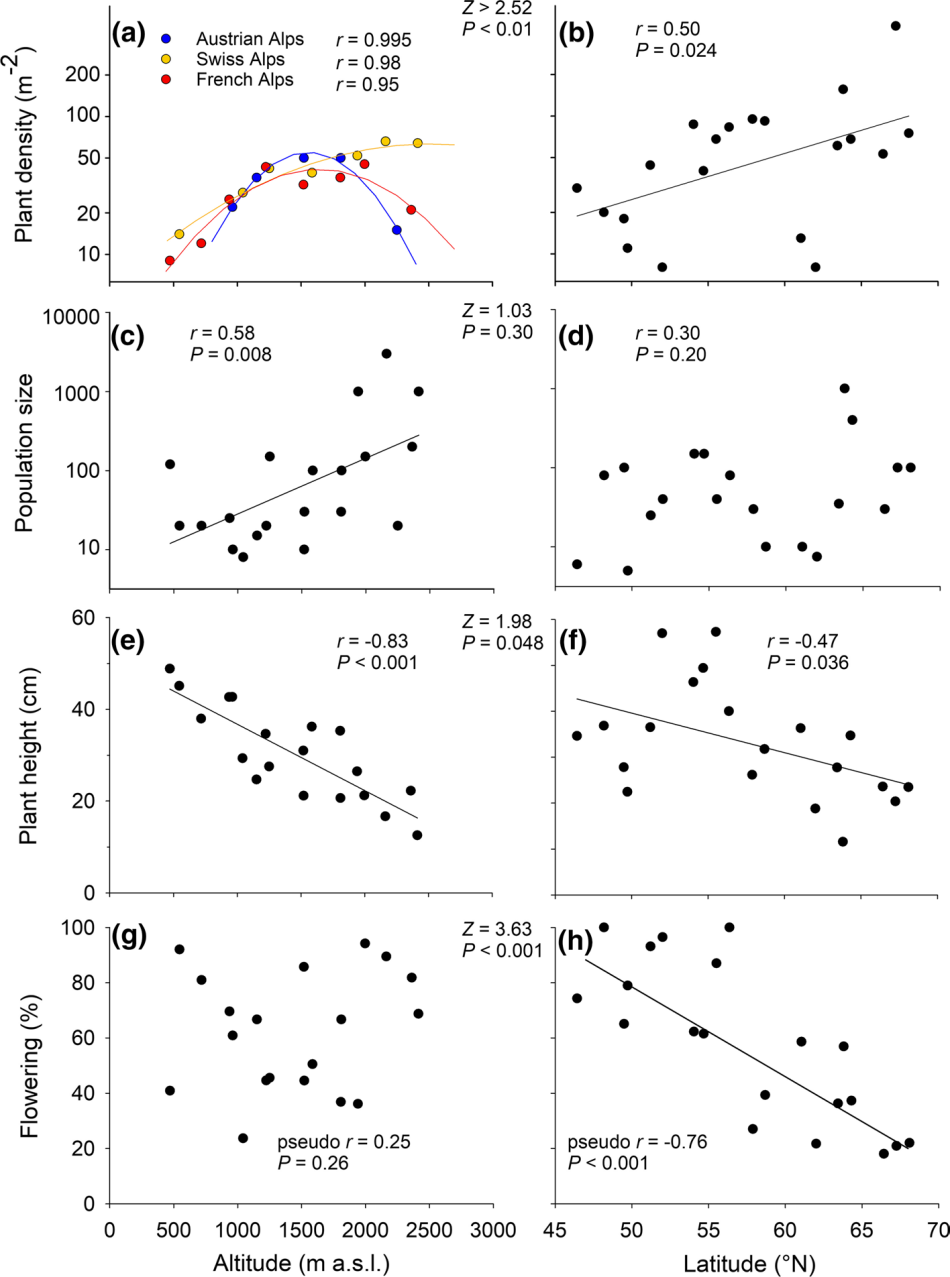
Relationships between characteristics of populations of *A. vulneraria* with altitude and latitude. For the altitudinal gradient, the effects of the three regions within the Alps were significant only in the case of maximum density (**a**) resulting in distinct models for each region. The results of *Z*-tests shown in between the panels indicate the significance of differences in the two correlation coefficients showing the strength of the relationship between a population trait and altitude or latitude. The *Z*-tests between the correlation coefficients of maximum density and altitude or latitude were calculated for the three regions separately

The relationship between maximal plant density and altitude was best fitted by quadratic functions (Fig. 2a, Supplementary Material Table S4). While in the Austrian and French Alps these functions indicated that plant density was highest at c. 1700 m, in Switzerland plant density increased up to 2500 m. However, the decline in density above 1800 m in Austria and above 2000 m in France was in both regions due to only a single population. Maximum plant density in the populations increased with latitude (Fig. 2b). In the 40 studied populations the number of plants and the maximum density were positively correlated (*r* = 0.43, *P* < 0.01). Population size varied along the altitudinal gradient from 80 to 30,000 individuals and along the latitudinal gradient from 50 to 10,000. The size of the populations increased significantly with altitude (Fig. 2c) but not with latitude (Fig. 2d). However, the difference in the strength of the relationships was not significant.

**Fig. 3:**
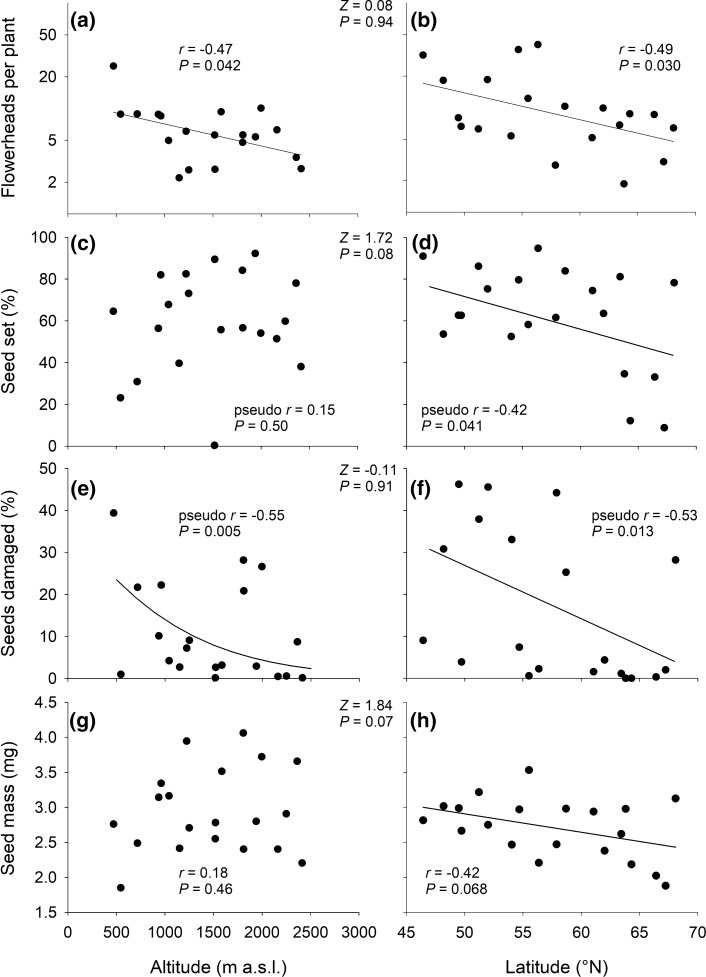
Relationships between mean reproductive traits in populations of *A. vulneraria* with altitude and latitude. A regression line was added if *P* < 0.1. The results of *Z*-tests shown in between the panels indicate the significance of differences in the two correlation coefficients showing the strength of the relationship between a population trait and altitude or latitude

Plant height decreased with both altitude and latitude indicating that both gradients were limiting plant growth, but altitude was a much better predictor (Fig. 2e, f). In contrast, the proportion of flowering plants did not vary consistently with altitude (Fig. 2g), but strongly decreased with latitude (Fig. 2h). The number of flowerheads per plant decreased from c. nine in the low altitude to four in the high altitude populations (Fig. 3a) and from 17 in the Central European to five in the arctic populations (Fig. 3b). The mean number of flowerheads per plant was related to mean plant size in the 40 populations as it increased with plant height (*r* = 0.52, *P* < 0.001).

Seed set decreased with latitude (Fig. 3d), but there was no relationship with altitude (Fig. 3c). However, the difference between the two correlation coefficients was only significant at the 0.08 level. The proportion of seeds damaged by herbivory decreased with both altitude and latitude (Fig. 3e, f). We detected eggs of *Cupido minimus* in 27 of the 40 sites but did not find any significant relationships between the probability of their presence and altitude (*P* = 0.96), latitude (*P* = 0.50), population size (Alt.: *P* = 0.42; Lat.: *P* = 0.10) or density of *A. vulneraria* (Alt.: *P* = 0.66; Lat.: *P* = 0.19). There was no clear relationship between mean seed mass and altitude (Fig. 3g), while there was some support (*P* = 0.068) that seed mass decreased with latitude (Fig. 3h). The difference between the two correlation coefficients was only significant at the 0.07 level.

### Influences of habitat characteristics on mean population traits

**Fig. 4:**
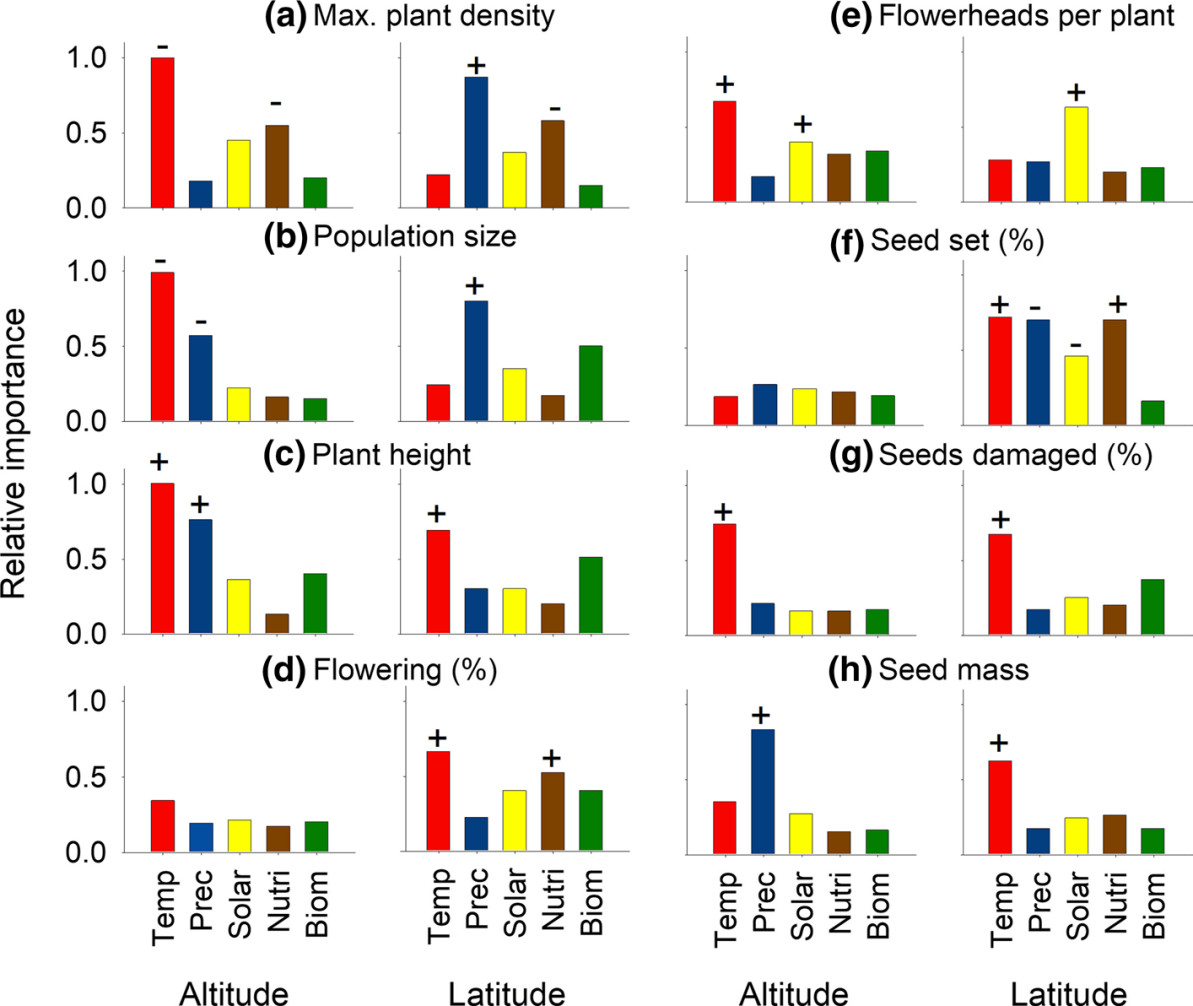
The effects of annual mean temperature (Temp), annual precipitation (Prec), solar radiation (Solar), PC soil nutrients (Nutri), and standing biomass (Biom) on mean population characteristics of *Anthyllis vulneraria* along an altitudinal and a latitudinal gradient. Given are importance values from averaging over all possible models including these variables. For variables that are part of the best model with the lowest AICc (Supplementary Material Table S6) the sign of the regression coefficient is shown: + , positive; −, negative effect. For model details see Supplementary Material Table S5

Annual mean temperature was the environmental factor that was most important in the models for six out of eight mean population traits along the altitudinal gradient (Fig. 4, Supplementary Material Table S5). Along the latitudinal gradient, temperature had also the highest importance values for most traits. Precipitation had overall the second highest importance. In the best models with the lowest AICc (Supplementary Material Table S6) mean plant performance in terms of growth and reproduction declined in parallel with temperature along both gradients, but density and population size increased with lower temperatures in the higher regions of the Alps. Precipitation, soil nutrients and solar radiation were further environmental factors that influenced some plant traits, although mostly in addition to temperature while standing biomass was never part of the best model.

## Discussion

We studied mean population traits of *A. vulneraria* along a latitudinal gradient that extended from the centre of the distribution of the species in Central Europe to its northern distributional margin, and along an altitudinal gradient that ranged from the lowlands to the altitudinal limit of the species in the Alps. Plant size, reproduction and density reacted similarly to changes in altitude and latitude and showed clinal variation, potentially allowing to assess the response of populations to predicted warming of the climate. However, some reproductive components like seed set and mass and the proportion of plants flowering responded differently to the two gradients and decreased with latitude but not with altitude indicating that the effects of climate warming on plant populations are likely to differ along the two gradients.

The abundant centre model predicts that all vital rates of a species like survival, reproduction, growth and recruitment are highest at the centre of the distribution of a species, where environmental conditions are assumed to be most suitable and decline towards the periphery (Brown 1984; Pironon et al. 2017). The reduced size and reproduction of *A. vulneraria* in peripheral northern and alpine populations were in line with the decline in plant performance towards the periphery predicted by the ACM (see e.g. also Jump and Woodward 2003; Angert 2006; Vaupel and Matthies 2012). However, only 43% of the studies reviewed by Pironon et al. (2017) found a significant decline of reproduction towards the periphery, while a further 21% found no clear pattern. In contrast to size and reproduction, the density of *A. vulneraria* increased along both the altitudinal and latitudinal gradients, indicating that recruitment must be much higher in peripheral high altitude and high latitude populations to more than compensate for the reduced reproduction. The higher recruitment in peripheral populations could be the result of higher survival of young plants due to the higher precipitation and the lower intraspecific competition between the smaller plants in these populations (see also Villellas et al. 2013 for another short-lived species). The opposite trends in vital rates indicate the existence of demographic compensation between reproduction and recruitment in populations of *A. vulneraria.* Such compensatory changes in vital rates across the range are thought to allow species to occupy larger areas of distribution (Doak and Morris 2010; Villellas et al. 2015). Our results thus contribute to the increasing evidence that in contrast to the predictions of the ACM demographic compensation may be common in widespread species (Villellas et al. 2013, 2015; Peterson et al. 2018).

### Plant size and density

The size of plants as measured by their height and their number of flowerheads decreased with both altitude and latitude. A similar pattern has been found in many other species (e.g. Woodward 1986; Weber and Schmid 1998; Hargreaves et al. 2015). In contrast, in some forest herbs plant height increased with latitude (De Frenne et al. 2011; Acharya et al. 2017). For *A. vulneraria*, the results of the multiple regressions indicated that the reduced size of plants with increasing altitude and latitude is mainly due to the decrease in temperature. Temperature is an important constraint to growth and plant size is known to decrease with temperature and the resulting shorter growing season in many species (Körner 2003; De Frenne et al. 2013).

In contrast to plant size, plant density increased with both altitude and latitude. This could be an effect of reduced intraspecific competition due to the smaller size of plants. Furthermore, the density of *A. vulneraria* increased with lower soil nutrient levels along both gradients. *Anthyllis vulneraria* is a poor competitor (Sterk 1975) and negatively affected by high nutrient levels due to increased competition by other species (Ellenberg et al. 1992).

### Seed set and seed mass

The lower seed set and reduced seed mass in high latitude populations of *A. vulneraria* could be due to several factors. First, plant size in *A. vulneraria* decreased with latitude and smaller plants often have lower seed set and smaller seeds due to resource limitation (Matthies 1990). In *A. vulneraria* the decrease in seed set with latitude was related to lower soil nutrients and temperatures, in line with the result of other studies (Moles and Westoby 2003; Wu et al. 2018). Second, the diversity and abundance of insect pollinators are reduced at high latitudes and dominated by Diptera (Elberling and Olesen 1999; Fulkerson et al. 2012). Because the flowers of *A. vulneraria* are exclusively pollinated by Hymenoptera (Couderc and Gorenflot 1978), seed set in the northern populations may have been lower due to a lack of pollen. Third, pollen limitation may result in increased self-fertilization and increased abortion of developing seeds and lower seed mass at higher latitudes.

While seed set and seed mass decreased with latitude, they did not vary consistently with altitude and temperature. As in other plant species (Körner et al. 1989; Guo et al. 2010) the size of plants of *A. vulneraria* declined with altitude, but this did not result in a decline of seed set and mass. Instead, both reproductive components were highly variable at all altitudes, confirming that local environmental variation may strongly influence reproduction in *A. vulneraria* (Kesselring et al. 2015). Environmental factors may also interact with pollen availability to determine reproduction in alpine environments (Totland 2001). The few other studies that have investigated the relationship between seed set and altitude have found conflicting results (Totland and Birks 1996; Vaupel and Matthies 2012; Hargreaves et al. 2015). Similarly, studies that have investigated how mean seed mass varies with altitude in individual species found also no consistent pattern. In some species, seed mass increased (Holm 1994; Olejniczak et al. 2018), whereas in others it declined (Totland and Birks 1996; Pluess et al. 2005; Guo et al. 2010; Olejniczak et al. 2018), or showed no relationship with altitude (Holm 1994; Pluess et al. 2005; Vaupel and Matthies 2012; Olejniczak et al. 2018).

### Seed predation

The seeds of *A. vulneraria* are attacked by several species of specific insect herbivores, e.g. *Cupido minimus*, *Hypera trilineata*, *Tychius* sp., *Bruchophagus* sp. (Sterk et al. 1982). Pre-dispersal seed predation decreased both along the latitudinal and the altitudinal gradient, although population size increased with altitude and plant density increased along both gradients. Larger and denser plant populations are more likely to be found by specific seed predators and to sustain viable populations of them (Kéry et al. 2001; Colling and Matthies 2004; Vaupel and Matthies 2012). In contrast to our results, the level of seed predation would thus have been expected to increase along the two gradients. The lower seed predation at high elevations and latitudes is most likely due to low temperatures and short summers that negatively affect larval development and abundance (Alonso 1999; Hodkinson 2005; Lee and Kotanen 2015). Our results are in line with those of many other studies that found a decrease in seed predation with increasing altitude (Alonso 1999; Giménez‐Benavides et al. 2008; Buckley et al. 2019) and the few that studied effects of latitude (Vergeer and Kunin 2011; Lee and Kotanen 2015; but see Anstett et al. 2014). Our results indicate that the positive effects of increased density and larger population size of the host species *A. vulneraria* cannot compensate for the negative effects of the more severe weather conditions on the populations of the seed predators and support the notion of a general decline of the importance of insect herbivory with increasing latitude (Schemske et al. 2009).

### Population structure

*Anthyllis vulneraria* is usually considered a biennial, which in the first year produces a rosette that in the second year flowers, fruits and then dies (Sterk 1975). In the current study, the proportion of flowering plants decreased strongly along the latitudinal gradient. A possible explanation for the high proportion of vegetative plants in the North could be the higher recruitment due to higher soil moisture, leading to an increase in the density of young vegetative plants in combination with a high mortality of vegetative plants over winter (Villellas et al. 2013). Alternatively, plants in the North may stay in a vegetative state for more than one year because flowering is delayed because of lower solar radiation, temperatures and nutrient availability (Lacey 1988; Lempe et al. 2005, but see Vergeer and Kunin 2011). This would increase the proportion of non-flowering plants over multiple growing and germination seasons as they accumulate in the population (Lacey 1988; Becker et al. 2006). In contrast, although similar environmental changes could be expected along the altitudinal gradient, at high altitudes both populations with a low and a high proportion of flowering plants were found. This indicates heterogeneity in local conditions (e.g. snow cover and time of snow melt) at a smaller scale than the Worldclim data that override the effects of general trends in climatic and edaphic conditions. Heterogeneity of small-scale habitat conditions is particularly high in alpine habitats (Körner 2007).

## Conclusions

Our study of variation in population mean traits along two gradients from the centre of the distribution of *A. vulneraria* in Central Europe to its range margins in the subarctic North and at the high altitudes in the Alps only partially support the abundant centre model (ACM). The reduced size and reproduction of *A. vulneraria* in peripheral populations were in line with the decline in performance towards the range margins predicted by the ACM. However, the increase of recruitment and plant density towards the range limits contributes to the growing evidence that the general decline in vital rates towards the periphery assumed by the ACM is too simplistic (Abeli et al. 2014; Pironon et al. 2017). Instead, demographic compensation between vital rates, as between reduced reproduction and increased recruitment in *A. vulneraria*, may be an important factor contributing to the large area of distribution and wide altitudinal range of a species (Doak and Morris 2010). The plasticity of the life cycle might also provide some buffering for widespread species against negative effects of climate change (Doak and Morris 2010; Villellas et al. 2015; Peterson et al. 2018, but see Sheth and Angert 2018).

Population characteristics of *A. vulneraria* varied strongly along the altitudinal and latitudinal gradients and environmental conditions influenced mean population traits, population structure and demography. Changes in temperature along the gradients appeared to have by far the strongest effects on the populations, followed by those in other climate variables like precipitation and solar radiation, and in soil nutrients. Increasing altitude and latitude both reduced size-related traits of plants and seed predation, but only with latitude was there a clinal decline in the proportion of plants flowering, and in seed set and seed mass. Observed latitudinal patterns in population characteristics are thus of only limited value to predict changes with altitude. This indicates that it will be more difficult to draw conclusions about potential impacts of future climate warming on plant populations in mountains, because of large local variation in important traits not related to altitude and annual mean temperature, but to small-scale variation in environmental conditions (Scherrer and Körner 2010; Oldfather and Ackerly 2019) and potentially in plant-pollinator interactions (Totland and Birks 1996). However, the actual responses of species to predicted climate warming will also depend on the genetic variation among populations and their phenotypic plasticity (Peterson et al. 2018). Our study did not allow us to disentangle the effects of phenotypic plasticity, genetic variation and local adaptation. Common garden experiments and molecular genetic analyses will be necessary to identify the mechanisms involved.

## Supplementary Information

Below is the link to the electronic supplementary material.

Supplementary file1 (PDF 560 kb)
