# Supplementary material for: Altitude and latitude have different effects on population characteristics of the widespread plant Anthyllis vulneraria
Source: Oecologia. 2021 Oct 2;197(2):537–49. doi: 10.1007/s00442-021-05030-6 (PMC8505396; doi:10.1007/s00442-021-05030-6)
Supplement: Supplementary file 1 — Supplementary file1 (PDF 560 kb) [file 442_2021_5030_MOESM1_ESM.pdf]

Supplementary Material

Altitude and latitude have different effects on population characteristics of the widespread plant *Anthyllis vulneraria*

Laura Daco, Guy Colling and Diethart Matthies

Corresponding author: Laura Daco, ldaco@mnhn.lu, +352 46 22 40 201

Table S1: *Anthyllis vulneraria* study sites.

Population	Country	Latitude (°N)	Longitude (°E)	Altitude (m a.s.l.)
**Altitudinal gradient**				
AFr1	France	45.05330	6.38920	2362
AFr2	France	45.05119	6.35326	1997
AFr3	France	45.15615	6.42366	1518
AFr4	France	45.21658	6.32499	1223
AFr5	France	45.09324	5.78040	471
AFr6	France	45.17351	6.03890	936
AFr7	France	45.12095	5.98519	717
AFr8	France	45.05978	6.31574	1807
ACh1	Switzerland	46.13377	7.05947	545
ACh2	Switzerland	46.08331	7.12650	1042
ACh3	Switzerland	46.04960	7.95643	2162
ACh4	Switzerland	46.25389	7.27336	1585
ACh5	Switzerland	46.27354	7.23742	1250
ACh6	Switzerland	46.08813	7.40669	1940
ACh7	Switzerland	46.10807	7.58007	2413
AAt1	Austria	47.39796	11.26613	961
AAt2	Austria	47.44211	11.65014	1521
AAt3	Austria	47.16064	11.71487	1810
AAt4	Austria	47.16901	11.35334	1151
AAt5	Austria	47.31265	11.38936	2250
**Latitudinal gradient**				
L1	France	46.43682	4.75282	323
L2	France	48.18803	5.55341	443
L3	Luxembourg	49.49562	5.99690	342
L4	Luxembourg	49.73139	6.28194	355
L5	Germany	51.22280	9.76097	442
L6	Germany	52.00507	10.40749	191
L7	Germany	54.04430	10.22901	32
L8	Germany	54.68729	9.43418	22
L9	Denmark	55.51500	9.42435	42
L10	Sweden	56.36709	12.80019	81
L11	Sweden	57.88923	11.94657	24
L12	Sweden	58.69792	11.21994	5
L13	Norway	61.06211	10.39708	438
L14	Norway	62.01385	9.20743	483
L15	Norway	63.44094	10.65668	18
L16	Iceland	63.81644	-22.69699	20
L17	Norway	64.31624	12.34754	168
L18	Sweden	66.42609	16.85014	453
L19	Norway	67.25107	15.42817	6
L20	Norway	68.10215	16.37830	47

Table S2: Pairwise correlations between the predictor variables used in the general and generalized linear models for both the altitudinal and latitudinal gradients. (*) $P < 0.1$; * $P < 0.05$; ** $P < 0.01$; *** $P < 0.001$.

	Annual mean temperature		Annual precipitation	Solar radiation		Standing biomass
	r		*r*	*r*		*r*
**Altitude**						
Annual precipitation	-0.628	**				
Solar radiation	-0.317		0.284			
Standing biomass	0.334		-0.087	0.002		
PC Soil nutrients	-0.103		0.130	0.057		-0.203
**Latitude**						
Annual precipitation	0.044					
Solar radiation	0.815	***	-0.258			
Standing biomass	-0.324		-0.021	-0.456	*	
PC Soil nutrients	0.539	*	0.040	0.666	**	-0.271

Table S3: Medians and ranges of habitat characteristics for the 40 studied *A. vulneraria* populations.

Habitat characteristic	Median	Range		
Exposure (° deviating from north)	114.5	0.0	-	177.0
Slope (%)	24.0	0.0	-	102.7
Standing biomass (g m^{-2})	96.1	23.0	-	334.2
pH	7.1	4.7	-	7.6
P_2O_5 (mg [100 g soil]$^{-1}$)	3.0	1.0	-	10.0
K_2O (mg [100 g soil]$^{-1}$)	8.0	2.0	-	40.0
C_{org} (%)	3.2	0.2	-	17.5
N_{tot} (%)	0.2	0.0	-	1.1
Annual mean temperature (°C)	6.1	-1.2	-	10.8
Annual precipitation (mm)	1037.5	413.0	-	1462.0

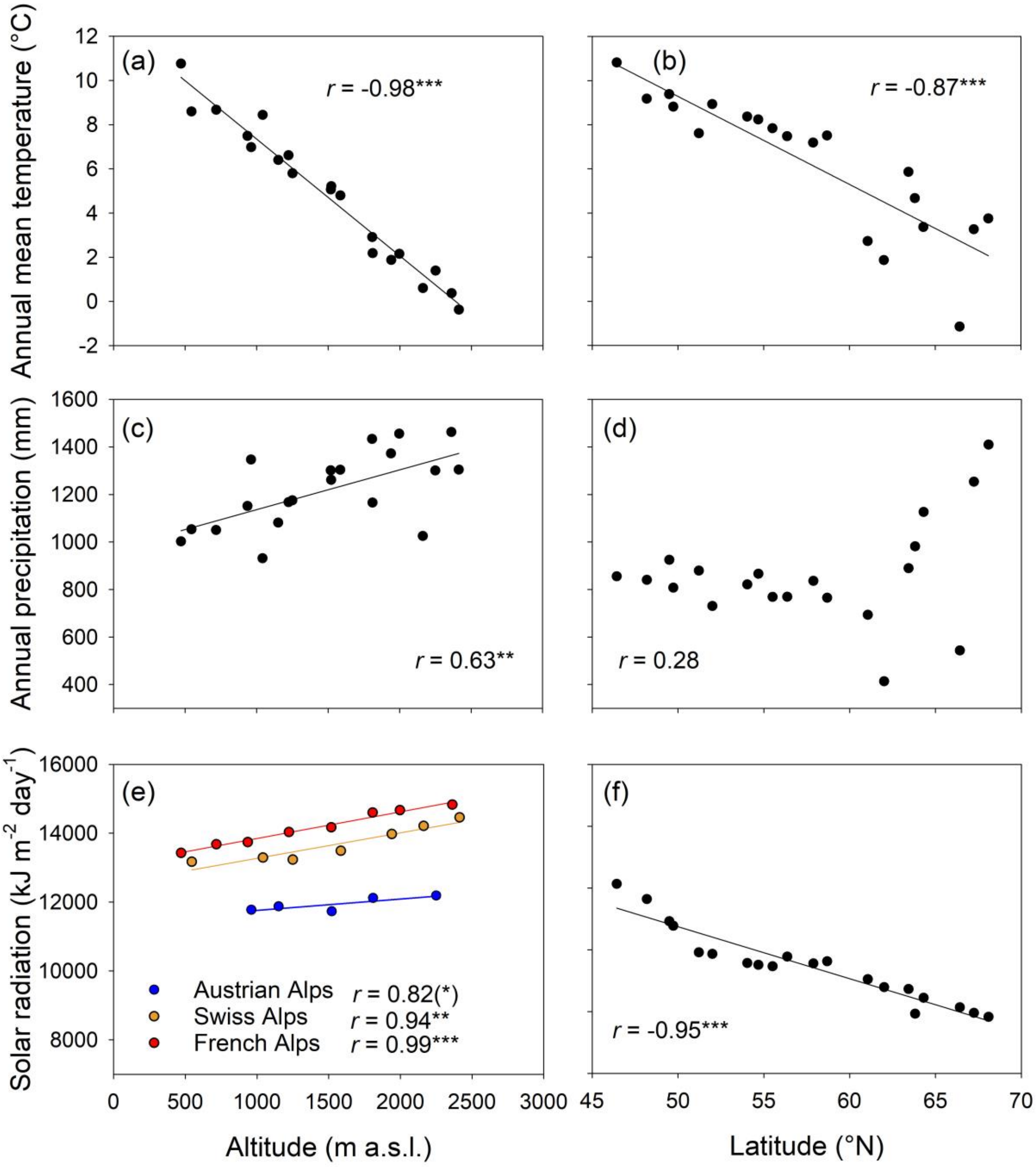

Fig. S1: Relationship between various habitat characteristics of *A. vulneraria* populations and (a, c, e) altitude for the populations along the altitude gradient and (b, d, f) latitude for the populations along the latitudinal gradient.

Table S4: Analysis of variance of the effects of the three regions within the Alps and the linear and quadratic effects of altitude on the maximal plant density of the populations of the altitudinal gradient. r^2 of the overall model = 0.94.

Max. plant density (m^{-2})	df	*F*	*P*
Region	2	13.2	0.001
Altitude linear	1	57.4	<0.001
Altitude quadratic	1	59.9	<0.001
Region x Alt. linear	2	8.7	0.005
Region x Alt. quadratic	2	11.6	0.002
Error	11		

Table S5: Results of model averaging of the effects of five explanatory variables on various population characteristics along the altitudinal and latitudinal gradients. Model averaging of all possible models and importance calculation was done using the R-package MuMIn. n: number of populations included in the analyses.

Dependent variable	Intercept	Explanatory variable	Importance	Estimate	Standard error
**Altitude (n = 19)**					
Max. plant density	2.301	Annual mean temperature	1.00	-0.06107	0.01422
		PC Soil nutrients	0.55	-0.06304	0.03313
		Solar radiation	0.45	-0.00007	0.00004
		Standing biomass	0.20	-0.00048	0.00054
		Annual precipitation	0.18	-0.00024	0.00031
Population size	4.581	Annual mean temperature	0.99	-0.18888	0.05458
		Annual precipitation	0.57	-0.00183	0.00096
		Solar radiation	0.22	0.00014	0.00014
		PC Soil nutrients	0.16	0.06067	0.11628
		Standing biomass	0.15	-0.00074	0.00184
Plant height	-18.640	Annual mean temperature	1.00	3.14337	0.62758
		Annual precipitation	0.76	0.02335	0.00986
		Standing biomass	0.40	0.02837	0.01749
		Solar radiation	0.36	0.00216	0.00141
		PC Soil nutrients	0.13	-0.41895	1.21362
Prop. flowering	0.223	Annual mean temperature	0.34	-0.10809	0.07935
		Solar radiation	0.21	0.00016	0.00022
		Standing biomass	0.20	0.00208	0.00338
		Annual precipitation	0.19	-0.00086	0.00160
		PC Soil nutrients	0.17	0.04993	0.22627
Flowerheads per plant	0.074	Annual mean temperature	0.67	0.04241	0.02033
		Solar radiation	0.40	0.00010	0.00006
		Standing biomass	0.34	-0.06627	0.05016
		PC Soil nutrients	0.32	0.00109	0.00079
		Annual precipitation	0.17	-0.00003	0.00048
Prop. seed set	0.541	Annual precipitation	0.26	0.00195	0.00177
		Solar radiation	0.24	-0.00028	0.00029
		PC Soil nutrients	0.21	0.21317	0.26602
		Standing biomass	0.19	-0.00177	0.00314
		Annual mean temperature	0.18	-0.03240	0.08476
Prop. seeds damaged	-3.219	Annual mean temperature	0.74	0.26420	0.12110
		Annual precipitation	0.21	0.00057	0.00369
		Standing biomass	0.17	-0.00078	0.00405
		Solar radiation	0.16	-0.00009	0.00036
		PC Soil nutrients	0.16	-0.02075	0.39170
Seed mass	-0.092	Annual precipitation	0.83	0.00231	0.00099
		Annual mean temperature	0.35	0.06893	0.05750
		Solar radiation	0.27	0.00016	0.00015
		Standing biomass	0.16	0.00052	0.00183
		PC Soil nutrients	0.15	-0.03694	0.12351

**Latitude (n = 20)**					
Max. plant density	1.341	Annual precipitation	0.87	0.00111	0.00040
		PC Soil nutrients	0.58	-0.26629	0.13404
		Solar radiation	0.37	-0.00017	0.00014
		Annual mean temperature	0.22	0.02906	0.06075
		Standing biomass	0.15	-0.00024	0.00197
Population size	2.207	Annual precipitation	0.80	0.00142	0.00056
		Standing biomass	0.50	0.00431	0.00244
		Solar radiation	0.35	-0.00025	0.00022
		Annual mean temperature	0.24	0.06261	0.08813
		PC Soil nutrients	0.17	-0.03557	0.23627
Plant height	40.272	Annual mean temperature	0.69	2.36622	1.35573
		Standing biomass	0.51	-0.09715	0.05568
		Annual precipitation	0.30	-0.01613	0.01392
		Solar radiation	0.30	-0.00296	0.00602
		PC Soil nutrients	0.20	-2.84218	4.96278
Prop. flowering	-3.557	Annual mean temperature	0.66	0.27155	0.12482
		PC Soil nutrients	0.52	0.90605	0.51620
		Solar radiation	0.40	0.00070	0.00052
		Standing biomass	0.40	0.00793	0.00497
		Annual precipitation	0.22	-0.00080	0.00114
Flowerheads per plant	0.111	Solar radiation	0.63	0.00015	0.00008
		Annual mean temperature	0.28	0.02761	0.04028
		Annual precipitation	0.27	-0.00038	0.00037
		Standing biomass	0.23	-0.00148	0.00174
		PC Soil nutrients	0.20	0.04763	0.15840
Prop. seed set	4.500	Annual mean temperature	0.72	0.24958	0.14977
		PC Soil nutrients	0.70	1.08700	0.60205
		Annual precipitation	0.70	-0.00249	0.00136
		Solar radiation	0.46	-0.00074	0.00058
		Standing biomass	0.16	-0.00384	0.00617
Prop. seeds damaged	-3.060	Annual mean temperature	0.67	0.28989	0.15632
		Standing biomass	0.37	-0.01380	0.00937
		Solar radiation	0.25	0.00010	0.00047
		PC Soil nutrients	0.20	0.37806	0.54226
		Annual precipitation	0.17	0.00098	0.00190
Seed mass	2.415	Annual mean temperature	0.62	0.06845	0.03673
		PC Soil nutrients	0.26	0.15930	0.16620
		Solar radiation	0.24	0.00002	0.00015
		Annual precipitation	0.17	0.00009	0.00045
		Standing biomass	0.17	-0.00029	0.00207

Table S6: Standardized regression coefficients for general and generalized linear models of the relationship between population characteristics of *Anthyllis vulneraria* along an altitudinal and a latitudinal gradient and habitat characteristics. Standardized regression coefficients for binomial models for proportions are calculated with the latent-theoretical method (Grace et al. 2018). t-values are given for variables with a gaussian distribution and z-values for the ones with a quasibinomial distribution. The best model was chosen using the lowest AICc for the variables with a gaussian distribution and using the lowest QAICc for the variables with a quasibinomial distribution using the R-package MuMIn. Only dependent variables are presented for which the best model contained at least one explanatory variable in addition to the intercept. Total r^2 is indicated for the models with a gaussian distribution, McFadden's pseudo r^2 for the ones with a quasibinomial one. Annual mean temperature is highlighted in bold face. * $P < 0.05$; ** $P < 0.01$; *** $P < 0.001$

Dependent variable	Explanatory variable	Coefficient	t/z-value	*P*
**Altitude (n = 19)**				
Log Max. plant density (r^2 = 0.62***)	**Annual mean temperature**	-0.76	-4.90	< 0.001
	PC Soil nutrients	-0.29	-1.85	0.083
Log Population size (r^2 = 0.58***)	**Annual mean temperature**	-0.94	-4.57	< 0.001
	Annual precipitation	-0.40	-1.92	0.073
Plant height (r^2 = 0.69***)	**Annual mean temperature**	1.06	6.65	< 0.001
	Annual precipitation	0.39	2.48	0.025
Log Flowerheads per plant (r^2 = 0.35**)	**Annual mean temperature**	0.63	2.83	0.012
	Solar radiation	0.41	1.83	0.086
Prop. seeds damaged (pseudo r^2 = 0.34)	**Annual mean temperature**	0.40	2.57	0.020
Seed mass (r^2 = 0.27*)	Annual precipitation	0.52	2.53	0.022
**Latitude (n = 20)**				
Log Max. plant density (r^2 = 0.49***)	Annual precipitation	0.58	3.33	0.004
	PC Soil nutrients	-0.43	-2.47	0.024
Log Population size (r^2 = 0.28*)	Annual precipitation	0.53	2.67	0.016
Plant height (r^2 = 0.24*)	**Annual mean temperature**	0.49	2.38	0.028
Prop. flowering (pseudo r^2 = 0.57)	**Annual mean temperature**	0.33	2.44	0.026
	PC Soil nutrients	0.28	1.95	0.068
Log Flowerheads per plant (r^2 = 0.28*)	Solar radiation	0.53	2.64	0.017
Prop. seed set (pseudo r^2 = 0.59)	**Annual mean temperature**	0.53	2.98	0.009
	Annual precipitation	-0.35	-2.89	0.011
	Solar radiation	-0.57	-2.53	0.023
	PC Soil nutrients	0.47	2.56	0.022
Prop. seeds damaged (pseudo r^2 = 0.30)	**Annual mean temperature**	0.46	2.36	0.030
Seed mass (r^2 = 0.23*)	**Annual mean temperature**	0.48	2.31	0.033
